# High-Frequency Oscillation Suppression Strategy for VSG MMC-HVDC Integrated Offshore Wind Farms Considering Frequency Coupling

**DOI:** 10.3390/s26051484

**Published:** 2026-02-26

**Authors:** Haichang Sun, Weiwei Yao, Hailang Shi, Liang Qin, Youhan Deng, Kaipei Liu

**Affiliations:** 1 School of Electrical Engineering and Automation, Wuhan University, Wuhan 430072, China; haichangsun@whu.edu.cn (H.S.); 2025282070123@whu.edu.cn (H.S.); kpliu@whu.edu.cn (K.L.); 2 Laboratory of Hydro-Wind-Solar Multi-Energy Control Coordination, Wuhan 430000, China; yao_weiwei@ctg.com.cn (W.Y.); deng_youhan@cypc.com.cn (Y.D.); 3 Institute of Science and Technology, China Three Gorges Corporation, Beijing 101100, China

**Keywords:** high-frequency oscillation, frequency coupling, VSG control, MMC, offshore wind farms, suppression strategy

## Abstract

**Highlights:**

**What are the main findings?**

- A significant high-frequency-range frequency coupling between the GSC and the VSG-controlled WFMMC has been discovered, which critically reshapes their positive-sequence impedances.
- Based on the identified key control loops responsible for frequency coupling, a high-frequency oscillation suppression strategy incorporating a band-stop filter in the WFMMC voltage sampling loop is proposed, with consideration of frequency coupling.

**What are the implications of the main findings?**

- This finding compels a revision of conventional stability theory by revealing that frequency coupling under VSG control extends into the high-frequency range, thereby mandating its incorporation into the design of high-frequency oscillation suppression strategies, unlike the conventional V-f control.
- The proposed strategy effectively suppresses the frequency coupling between the GSC and the VSG-controlled WFMMC, thereby mitigating high-frequency oscillations in the system induced by such coupling under various challenging operating conditions.

**Abstract:**

In MMC-HVDC-integrated offshore wind farms, the Wind-Farm-side MMC (WFMMC) is increasingly adopting Virtual Synchronous Generator (VSG) control to provide active support. However, this control strategy may introduce high-frequency oscillations that cannot be predicted by conventional stability analysis. Existing suppression strategies, designed for WFMMC under conventional V-f control, fail to account for frequency coupling effects in the high-frequency range, making it difficult to effectively analyze or suppress such oscillations. To address this issue, this article reveals a significant high-frequency-range frequency coupling effect between the wind turbine’s Grid-Side Converter (GSC) and the VSG-controlled MMC, which is distinct from systems with conventional V-f control. It is further identified that the asymmetric control structure introduced by the VSG control and control delay are the key factors driving this coupling. Based on this finding, an oscillation suppression strategy incorporating a band-stop filter in the WFMMC voltage sampling loop is proposed. Time-domain simulations demonstrate the effectiveness of this strategy under various operating conditions.

## 1. Introduction

With the growing severity of global warming and the fossil fuel crisis, the development and utilization of renewable energy sources, such as wind power, have become a focal point of the global energy transition [1,2]. Compared to onshore wind power, offshore wind power offers advantages including a smaller footprint, higher wind speeds, and greater power generation capacity [3], leading to a remarkably rapid development and commissioning trend in recent years. Over ten offshore wind power integration projects via flexible High Voltage Direct Current (HVDC) have been completed and put into operation in Europe and China, such as BorWin1 [4], with more than 30 additional projects under planning or construction [5]. The Modular Multilevel Converter (MMC), with its benefits of lower harmonic distortion, high power capacity, and modular design, is widely adopted as the flexible HVDC converter for large-scale offshore wind power integration.

Since the sending-end system of offshore wind power integrated via MMC-HVDC is an islanded grid, the Wind Farm-side MMC (WFMMC) is responsible for establishing the voltage and frequency of this system. Currently, most WFMMCs in offshore wind power integration projects adopt the constant voltage and frequency control (V-f control) strategy [6,7], where the WFMMC directly provides the reference values for voltage magnitude and phase angle to ensure the normal operation of wind turbines. However, under this V-f control strategy, the MMC exhibits no active response or support capability to frequency and voltage disturbances within the sending-end system, resulting in poor disturbance rejection performance [8]. This presents a significant challenge for large-scale offshore wind power integration. Therefore, WFMMC control strategies based on Virtual Synchronous Generators (VSGs), which inherently possess frequency and voltage support capabilities, are poised to become the mainstream choice for future large-scale offshore wind power integration systems and have increasingly become a research focus in recent years [9,10,11,12]. Compared to V-f control, VSG control incorporates two additional control loops: virtual synchronous and virtual excitation [13,14]. This enables the WFMMC to actively provide active power support and suppress frequency deviations during power disturbances in the Alternating Current (AC) system [15,16]. Furthermore, it helps maintain a certain degree of voltage regulation capability during sudden AC voltage changes, thereby providing essential voltage support [17].

It is noteworthy that several high-frequency oscillation incidents have been reported in operational offshore wind power integration projects via MMC-HVDC [18,19]. The associated harmonic voltage and current components have led to severe consequences, including converter blocking trips, and filter burnout or explosions. Impedance-based analysis has proven to be an effective method for analyzing and suppressing such high-frequency oscillations. Numerous studies have developed impedance models based on theories such as Harmonic State Space (HSS) for key components, including Grid-Side Converters (GSCs) of wind turbines [20,21], MMCs under V-f control [22,23], and MMCs under VSG control [24]. These studies utilize the positive-sequence impedances of the GSCs and the MMCs to calculate phase margins for system stability assessment, analyze oscillation mechanisms, and subsequently design oscillation suppression strategies [25].

However, the stability of offshore wind power integration via MMC-HVDC can be significantly affected by frequency coupling [26]. The phenomenon of frequency coupling was first investigated in scenarios involving a “grid-MMC” system. Its fundamental principle can be summarized as follows: a positive-sequence disturbance from the MMC can be influenced by the MMC’s own nonlinear control loops and the negative-sequence impedance of the AC grid, thereby generating an additional negative-sequence response. This response, in turn, acts as a disturbance, causing changes in the MMC’s positive-sequence response. Ultimately, the frequency coupling effect manifests as an alteration in the MMC’s equivalent positive-sequence impedance. In the offshore wind power integration system, both primary components, the GSC and the MMC, are power electronic converters rather than constant grid impedances. Consequently, their positive- and negative-sequence coupling dynamics interact at the Point of Common Coupling (PCC). This results in a more complex frequency coupling process, where the equivalent positive-sequence impedances of both the GSC and the MMC can be modified. Such mutual impedance variation may degrade system stability, potentially leading to oscillation phenomena in systems deemed stable by conventional impedance analysis based solely on converter positive-sequence impedances, thereby contradicting theoretical predictions. Some existing studies [26,27] have conducted a preliminary analysis of the frequency coupling effect between GSCs and V-f-controlled MMCs. These studies suggest that the frequency coupling between the GSC and the MMC is primarily induced by asymmetric control structures of the GSC, such as the Phase-Locked Loop (PLL), and that this coupling is predominantly confined to the sub-/super-synchronous frequency range. Consequently, they conclude that frequency coupling can be neglected in the analysis and suppression of high-frequency oscillations.

Optimizing the controller parameters of the GSC and WFMMC to enhance the damping characteristics at the oscillation frequency is the most straightforward approach for oscillation suppression. For instance, Reference [28] considers the interactions between the GSC and the WFMMC and designs a parameter optimization scheme to achieve oscillation suppression. However, due to the presence of two types of converters, the number of control parameters to be optimized increases significantly compared to conventional scenarios, which substantially raises the difficulty of parameter coordination. Moreover, given the randomness and volatility of offshore wind power output, real-time optimization of control parameters is extremely challenging. Such optimization often requires system shutdown for parameter adjustment, leading to additional economic losses [29]. Based on the aforementioned conclusions, and under the premise of neglecting frequency coupling in the high-frequency range, studies such as [25,30,31] have proposed high-frequency oscillation suppression strategies by adding extra control branches to the MMC, such as virtual impedance or virtual admittance. Concurrently, Refs. [7,19,32] have designed a series of high-frequency oscillation suppression strategies by incorporating additional filtering into existing MMC control branches, including voltage feedforward and current feedback loops. While these strategies have demonstrated good performance when applied to systems with MMCs under V-f control, they have not yet been implemented in actual engineering projects employing VSG-controlled MMCs. Consequently, their effectiveness for oscillation suppression in such VSG MMC-HVDC-integrated offshore wind farms remains unverified.

In summary, existing research has primarily focused on the frequency coupling mechanism between GSCs and V-f-controlled MMCs, attributing the coupling mainly to control structures like PLL. It has not considered the impact of active-support control strategies, such as virtual synchronous control, on frequency coupling and overall system stability. This oversight could potentially lead to high-frequency oscillations induced by frequency coupling when MMCs adopt VSG control. Furthermore, existing studies suggest that frequency coupling between GSCs and MMCs is confined to the sub-/super-synchronous frequency range below 100 Hz, neglecting potential frequency coupling effects in the high-frequency range (hundreds to thousands of Hz). Consequently, the design of existing high-frequency oscillation suppression strategies for MMC-HVDC-integrated offshore wind farms does not account for frequency coupling, which may result in suppression strategies failing to achieve the intended control performance or even becoming ineffective, thereby rendering them unable to mitigate high-frequency oscillations.

Therefore, this article reveals the decisive role of active-support components, such as virtual synchronous and virtual excitation in VSG-controlled MMCs, on high-frequency-range frequency coupling effects. It is further demonstrated that the intensity of this coupling increases with control delay under VSG control. Building on this insight, an oscillation suppression strategy based on band-stop filtering in the MMC voltage sampling loop that accounts for these key frequency-coupling mechanisms is proposed to reduce this coupling and ensure system stability. The contributions of this article are categorized into three main aspects.

1. The significant high-frequency-range frequency coupling effect between the GSC and the VSG-controlled WFMMC, which is distinct from that with the V-f-controlled WFMMC, is revealed. It thereby explains the mechanism behind high-frequency oscillation occurring between the GSC and the VSG-controlled WFMMC that is not predicted by conventional stability analysis methods.
2. It is identified that the asymmetric control structure introduced by the VSG control and the time delay are the key factors responsible for the aforementioned high-frequency-range frequency coupling. This finding provides critical guidance for the design of an oscillation suppression strategy.
3. Based on the key influencing factors of frequency coupling, a high-frequency oscillation suppression strategy incorporating band-stop filtering in the MMC voltage sampling loop is proposed. This strategy significantly reduces the frequency coupling between the GSC and the WFMMC caused by VSG control and time delay, thereby effectively suppressing high-frequency oscillations in VSG MMC-HVDC-integrated offshore wind farms.

This article is organized as follows: In Section 2, impedance models for the wind turbine GSC and the WFMMC are established, and the high-frequency oscillation phenomena within the system, along with the limitations of conventional stability analysis, are introduced. In Section 3, the mechanism of frequency coupling between the GSC and WFMMC is analyzed, and the key factors influencing this coupling are examined. In Section 4, the proposed high-frequency oscillation suppression strategy is presented based on the key influencing factors. In Section 5, the effectiveness of the proposed strategy under various operating conditions is validated through simulation.

## 2. Impedance Modeling and Stability Analysis Misjudgment

### 2.1. Configuration of the System

**Figure 1:**
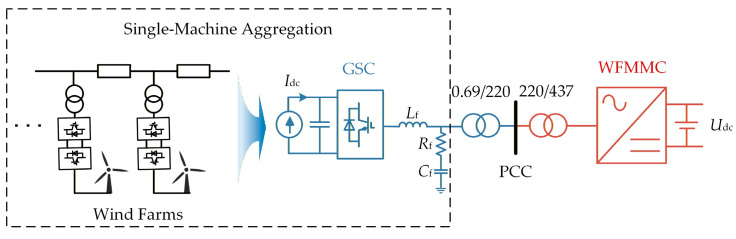
Configuration of the system studied.

The topology of the sending-end of the MMC-HVDC-integrated offshore wind farm studied in this article is shown in Figure 1. The offshore wind farm comprises 70 direct-drive wind turbines, each with a rated capacity of 10 MW. As this article focuses on the interaction between the GSCs and the WFMMC, the wind farm is aggregated into a single-machine equivalent model using the method in [33,34], where the machine-side dynamics of the turbines are simplified as a constant DC current source, *I*_dc_. *L*_f_, *R*_f_, and *C*_f_ represent the filter inductance, resistance, and capacitance of the GSC, respectively. The GSC is connected to the PCC via a 0.69/220 kV step-up transformer. The WFMMC has a rated power of 750 MW. Since the receiving-end MMC of the MMC-HVDC employs constant DC voltage control, which is assumed to be effective, the DC side of the WFMMC can be considered as a constant DC voltage source. The WFMMC is connected to the PCC through a 437/220 kV step-down transformer. Detailed electrical parameters of the system are provided in Appendix A Table A1.

### 2.2. Impedance Modeling and Verification of GSC and WFMMC

**Figure 2:**
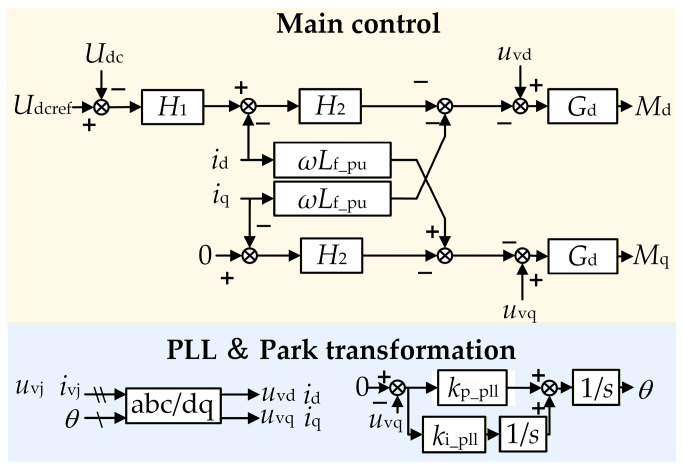
Control block diagram of the GSC.

The control structure of the GSC is shown in Figure 2. It employs a conventional grid-following control strategy. The reference phase *θ* for its PARK transformation is provided by a Phase-Locked Loop (PLL). *k*_p_pll_, *k*_i_pll_ are the proportional and integral coefficients of the PI controller in the PLL, respectively. *u*_vj_, *i*_vj_ (j = a, b, c) are the three-phase AC voltages and currents of the GSC. *u*_vd_, *u*_vq_ are the d-axis and q-axis voltages of the GSC, respectively. *i*_vd_, *i*_vq_ are the d-axis and q-axis currents of the GSC, respectively. *U*_dc_ and *U*_dcref_ are the sampled value and the reference value of the GSC DC voltage, respectively. The outer loop *H*_1_ utilizes constant DC voltage control and constant reactive power control (with the reactive power reference typically set to zero) to generate the current reference. This reference value is then fed into the inner current loop *H*_2_ to produce the modulation signals *M*_d_ and *M*_q_. *L*_f_pu_ is the per-unit value of the filter inductance, and *G*_d_ represents the delay of the GSC. The control parameters for the aforementioned loops are provided in Appendix A Table A2. It should be noted that the control parameters for the GSC in this article are given in per-unit values.

Since the impedance modeling of grid-following GSCs has been well established, this article considers the dynamics of key components—including the PLL and coordinate transformation, the DC voltage control loop, the inner current loop, as well as time delay and modulation—and directly presents the GSC impedance model in the dq-frame [23], as given in Equation (1).

(1) $$Z_{\mathrm{dqPMSG}}=\left[G_{\mathrm{fg}}+G_{\mathrm{de}}\left(G_{c}+G_{\mathrm{dg}}-G_{u}G_{c}G_{\mathrm{dc}}G_{\mathrm{dc},i}\right)\right]{\left\{I+G_{\mathrm{de}}\left[\left(G_{c}+G_{\mathrm{dg}}\right)G_{\mathrm{PLL}}^{i}+G_{\mathrm{PLL}}^{u}-G_{u}G_{c}G_{\mathrm{dc}}G_{\mathrm{dc},u}\right]\right\}}^{-1}$$

In the model, **G**_u_ and **G**_c_ represent the dynamics of the PI controllers for the DC voltage control loop (*H*_1_) and the current control loop (*H*_2_), respectively. **G**_de_ represents the control delay of the GSC. **G**_dg_ represents the dynamics of the decoupling loop within the current loop. $G_{\mathrm{PLL}}^{u}$ and $G_{\mathrm{PLL}}^{i}$ represent the dynamics arising from phase deviations in the voltage and current signals, respectively, due to the influence of the PLL. *G*_dc_, **G**_dc,u_, and **G**_dc,i_ represent the relationships between DC-side power, DC voltage, and current.

Based on Equations (2) and (3), the sequence impedance matrix **Z**_pnPMSG_ and the sequence admittance matrix **Y**_pnPMSG_ of the GSC can be further derived.

(2) $$Z_{\mathrm{pnPMSG}}=\frac{1}{2}\left[\begin{array}{cc} 1 & j\\ 1 & -j \end{array}\right]Z_{\mathrm{dqPMSG}}\left[\begin{array}{cc} 1 & 1\\ -j & j \end{array}\right]$$

(3) $$Y_{\mathrm{pnPMSG}}=Z_{\mathrm{pnPMSG}}^{-1}$$

Based on the admittance matrix **Y**_pnPMSG_ of the GSC, the relationship between the positive- and negative-sequence voltages and currents at its output port can be expressed as:

(4) $$\left[\begin{array}{c} \Delta i_{\mathrm{PMSG}}\left(s+j\omega_{0}\right)\\ \Delta i_{\mathrm{PMSG}}\left(s-j\omega_{0}\right) \end{array}\right]=\underset{Y_{\mathrm{pnPMSG}}}{\underset{︸}{\left[\begin{array}{cc} Y_{\mathrm{pp}} & Y_{\mathrm{pn}}\\ Y_{\mathrm{np}} & Y_{\mathrm{nn}} \end{array}\right]}}\left[\begin{array}{c} \Delta u_{\mathrm{PMSG}}\left(s+j\omega_{0}\right)\\ \Delta u_{\mathrm{PMSG}}\left(s-j\omega_{0}\right) \end{array}\right]$$

**Figure 3:**
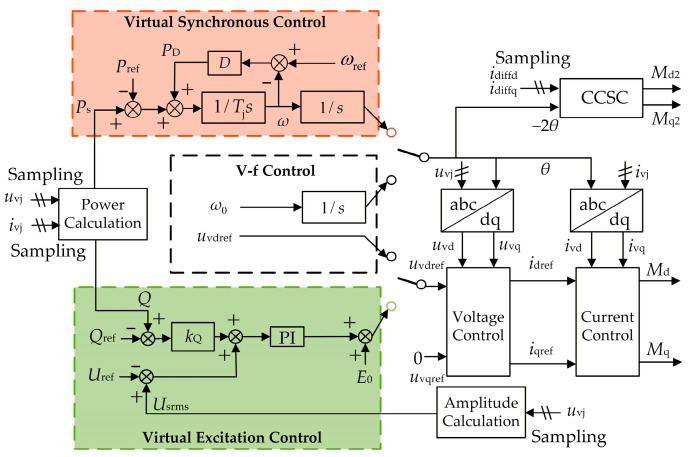
Control block diagram of the WFMMC.

The WFMMC primarily employs two typical control strategies: V-f control and VSG control, with their corresponding control block diagrams shown in Figure 3. The VSG control structure illustrated in Figure 3 represents the most widely adopted configuration in practical MMC-HVDC engineering projects [35], featuring the typical structure of virtual synchronous loop, virtual excitation loop and voltage/current control loops. While alternative VSG implementations exist [35], the selected structure focuses on the essential features that critically influence high-frequency oscillation characteristics.

Both strategies utilize dual-loop voltage and current control along with circulating current suppression control (CCSC). *i*_dref_ and *i*_qref_ represent the d-axis and q-axis current reference values for the inner current loop of the WFMMC, respectively. *i*_diffd_ and *i*_diffq_ are the d-axis and q-axis components of the WFMMC’s circulating current, respectively. *M*_d2_ and *M*_q2_ denote the d-axis and q-axis double-frequency modulation signals generated by the CCSC, respectively.

The key distinction lies in how the reference phase angle *θ* for the coordinate transformation and the d-axis voltage reference *u*_vdref_ are generated. Under V-f control, the reference phase angle *θ* is obtained by directly integrating the rated angular frequency *ω*_0_, and the d-axis voltage reference *u*_vdref_ is set directly to the rated AC voltage. In contrast, VSG control incorporates additional virtual synchronous and virtual excitation control loops. The reference phase angle is generated by the virtual synchronous loop, while the d-axis voltage reference is generated by the virtual excitation loop. *P*_s_ is the active power output by the WFMMC, and *P*_ref_ is its active power reference; *P*_D_ is the damping power of the WFMMC’s virtual inertia loop; *D* is the virtual inertia damping coefficient; *T*_j_ is the virtual inertia time constant. *Q* is the reactive power output by the WFMMC, and *Q*_ref_ is its reactive power reference; *U*_rms_ is the root mean square (RMS) value of the WFMMC’s output AC voltage; and *U*_ref_ is its RMS voltage reference; *k*_Q_ is the reactive power–voltage droop coefficient; *E*_0_ is the no-load voltage amplitude reference. The control parameters for the aforementioned loops are detailed in Appendix A Table A3. It should be noted that the control parameters for the WFMMC in this article are given in per-unit values.

This article establishes the impedance model of the WFMMC utilizing Harmonic State Space (HSS) theory. Due to space constraints, only the key steps of the impedance modeling process are presented here, along with the dynamics of key components such as virtual synchronous and virtual excitation. The detailed modeling procedure can be found in the authors’ previous work [36]. The HSS model of the MMC is divided into four parts:

(5) $$\;\left\{\begin{array}{l} \left(sI+N\right)\hat{X}=A_{\mathrm{Hx}}\hat{X}+A_{\mathrm{Hz}}\hat{Z}+B_{H}\hat{U}\\ \hat{Z}=C_{\mathrm{Hm}1}{\hat{M}}_{1}+C_{\mathrm{Hm}2}{\hat{M}}_{2}\\ {\hat{M}}_{1}=D_{H1}\hat{X}+E_{H1}\hat{U}\\ {\hat{M}}_{2}=D_{H2}\hat{X}+E_{H2}\hat{U}+F_{H2}{\overset{⌢}{I}}_{\mathrm{dq}} \end{array}\right.$$

The first and second equations in (5) are derived from the electrical system of the WFMMC, while the third and fourth equations are determined by its control system. Consequently, the transfer function matrix of the WFMMC is given by:

(6) $$\begin{array}{l} H_{\mathrm{MMC}}^{\mathrm{tf}}={\left(sI+N-A_{\mathrm{Hx}}-A_{\mathrm{Hz}}\left(C_{\mathrm{Hm}1}D_{H1}+C_{\mathrm{Hm}2}D_{H2}+C_{\mathrm{Hm}2}F_{H2}\right)\right)}^{-1}\cdot \\ \left(A_{\mathrm{Hz}}\left(C_{\mathrm{Hm}1}E_{H1}+C_{\mathrm{Hm}2}E_{H2}\right)+B_{H}\right) \end{array}$$

The sequence impedance matrix of the MMC, **Z**_pnMMC_, is given by:

(7) $$Z_{\mathrm{pnMMC}}={\left[\begin{array}{cc} H_{\mathrm{MMC}}^{\mathrm{tf}}\left(14,8\right) & H_{\mathrm{MMC}}^{\mathrm{tf}}\left(14,4\right)\\ H_{\mathrm{MMC}}^{\mathrm{tf}}\left(6,8\right) & H_{\mathrm{MMC}}^{\mathrm{tf}}\left(6,4\right) \end{array}\right]}^{-1}$$

For VSG-controlled WFMMC, the virtual rotor equation of the virtual synchronous control can be written as:

(8) $$\left\{\begin{array}{l} \frac{d\omega }{dt}=\frac{1}{T_{j}}\left(P-D\left(100\pi -\omega \right)\right)\\ \frac{d\theta }{dt}=\omega \end{array}\right.$$

where *T*_j_ represents the inertia time constant, *D* represents the damping coefficient, and *ω* represents the virtual angular velocity used in control.

The HSS form of Equation (8) can be expressed as:

(9) $$\begin{array}{l} G_{\mathrm{HPLL}}^{\prime }\hat{\theta }=\left[\begin{array}{cc} U_{\mathrm{Hvd}}^{c} & U_{\mathrm{Hvq}}^{c} \end{array}\right]\left[\begin{array}{c} {\hat{I}}_{d}^{c}\\ {\hat{I}}_{q}^{c} \end{array}\right]+\left[\begin{array}{cc} I_{\mathrm{Hd}}^{c} & I_{\mathrm{Hq}}^{c} \end{array}\right]\left[\begin{array}{c} {\hat{U}}_{\mathrm{vd}}^{c}\\ {\hat{U}}_{\mathrm{vq}}^{c} \end{array}\right]\\ =\left[\begin{array}{cc} U_{\mathrm{Hvd}}^{c} & U_{\mathrm{Hvq}}^{c} \end{array}\right]\left(\frac{P_{H}}{U_{\mathrm{base}}}\left[\begin{array}{c} {\hat{I}}_{a}\\ {\hat{I}}_{b}\\ {\hat{I}}_{c} \end{array}\right]+\left[\begin{array}{c} I_{\mathrm{Hq}}^{c}\\ -I_{\mathrm{Hd}}^{c} \end{array}\right]\hat{\theta }\right)+\left[\begin{array}{cc} I_{\mathrm{Hd}}^{c} & I_{\mathrm{Hq}}^{c} \end{array}\right]\left(\frac{P_{H}}{U_{\mathrm{base}}}\left[\begin{array}{c} {\hat{U}}_{\mathrm{va}}^{c}\\ {\hat{U}}_{\mathrm{vb}}^{c}\\ {\hat{U}}_{\mathrm{vc}}^{c} \end{array}\right]+\left[\begin{array}{c} U_{\mathrm{Hvq}}^{c}\\ -U_{\mathrm{Hvd}}^{c} \end{array}\right]\hat{\theta }\right)\\ =\left[\begin{array}{cc} U_{\mathrm{Hvd}}^{c} & U_{\mathrm{Hvq}}^{c} \end{array}\right]\frac{P_{H}}{U_{\mathrm{base}}}\left[\begin{array}{c} {\hat{I}}_{a}\\ {\hat{I}}_{b}\\ {\hat{I}}_{c} \end{array}\right]+\left[\begin{array}{cc} I_{\mathrm{Hd}}^{c} & I_{\mathrm{Hq}}^{c} \end{array}\right]\frac{P_{H}}{U_{\mathrm{base}}}\left[\begin{array}{c} {\hat{U}}_{\mathrm{va}}^{c}\\ {\hat{U}}_{\mathrm{vb}}^{c}\\ {\hat{U}}_{\mathrm{vc}}^{c} \end{array}\right] \end{array}$$

where $G_{\mathrm{HPLL}}^{\prime }=\mathrm{diag}\left(\cdots ,\left(s-j\omega_{1}\right)\left(T_{j}\left(s-j\omega_{1}\right)-D\right),s\left(T_{j}s-D\right),\left(s+j\omega_{1}\right)\left(T_{j}\left(s+j\omega_{1}\right)-D\right),\cdots \right)$.

The expression for $\hat{\theta }$ can be expressed as:

(10) $$\hat{\theta }={\left(G_{\mathrm{HPLL}}^{\prime }\right)}^{-1}\cdot \left[\begin{array}{cc} U_{\mathrm{Hvd}}^{c} & U_{\mathrm{Hvq}}^{c} \end{array}\right]\frac{P_{H}}{U_{\mathrm{base}}}\left[\begin{array}{c} {\hat{I}}_{a}\\ {\hat{I}}_{b}\\ {\hat{I}}_{c} \end{array}\right]+{\left({G^{\prime }}_{\mathrm{HPLL}}\right)}^{-1}\cdot \left[\begin{array}{cc} I_{\mathrm{Hd}}^{c} & I_{\mathrm{Hq}}^{c} \end{array}\right]\frac{P_{H}}{U_{\mathrm{base}}}\left[\begin{array}{c} {\hat{U}}_{\mathrm{va}}^{c}\\ {\hat{U}}_{\mathrm{vb}}^{c}\\ {\hat{U}}_{\mathrm{vc}}^{c} \end{array}\right]$$

Based on Figure 3, the small-signal dynamics of the virtual excitation control loop can be expressed as:

(11) $$\Delta U_{\mathrm{dref}}^{c}=\left(-\Delta U_{\mathrm{srms}}+k_{Q}\Delta Q\right)\left(k_{\mathrm{pE}}+k_{\mathrm{iE}}/s\right)$$

where $U_{\mathrm{srms}}=\sqrt{{\left(U_{\mathrm{vd}}^{c}\right)}^{2}+{\left(U_{\mathrm{vq}}^{c}\right)}^{2}}$

The HSS form of $\Delta U_{\mathrm{dref}}^{c}$ can be expressed as:

(12) $$\begin{array}{l} {\hat{U}}_{\mathrm{vdref}}^{c}=G_{\mathrm{PIec}}\cdot (-\left[\begin{array}{cc} \frac{U_{\mathrm{vd}}^{c}}{\sqrt{{\left(U_{\mathrm{vd}}^{c}\right)}^{2}+{\left(U_{\mathrm{vq}}^{c}\right)}^{2}}} & \frac{U_{\mathrm{vq}}^{c}}{\sqrt{{\left(U_{\mathrm{vd}}^{c}\right)}^{2}+{\left(U_{\mathrm{vq}}^{c}\right)}^{2}}} \end{array}\right]\left[\begin{array}{c} {\hat{U}}_{\mathrm{vd}}^{c}\\ {\hat{U}}_{\mathrm{vq}}^{c} \end{array}\right]\\ +k_{Q}\left[\begin{array}{cc} U_{\mathrm{Hvq}}^{c} & -U_{\mathrm{Hvd}}^{c} \end{array}\right]\left[\begin{array}{c} {\hat{I}}_{d}^{c}\\ {\hat{I}}_{q}^{c} \end{array}\right]+k_{Q}\left[\begin{array}{cc} -I_{\mathrm{Hq}}^{c} & I_{\mathrm{Hd}}^{c} \end{array}\right]\left[\begin{array}{c} {\hat{U}}_{\mathrm{vd}}^{c}\\ {\hat{U}}_{\mathrm{vq}}^{c} \end{array}\right])\\ =G_{\mathrm{PIec}}\cdot (\underset{G_{\mathrm{Heuv}}}{\underset{︸}{\left[\begin{array}{cc} \left(-\frac{U_{\mathrm{vd}}^{c}}{\sqrt{{\left(U_{\mathrm{vd}}^{c}\right)}^{2}+{\left(U_{\mathrm{vq}}^{c}\right)}^{2}}}-k_{Q}I_{q}^{c}\right)\cdot I_{h} & \left(-\frac{U_{\mathrm{vq}}^{c}}{\sqrt{{\left(U_{\mathrm{vd}}^{c}\right)}^{2}+{\left(U_{\mathrm{vq}}^{c}\right)}^{2}}}+k_{Q}I_{d}^{c}\right)\cdot I_{h} \end{array}\right]}}\left[\begin{array}{c} {\hat{U}}_{\mathrm{vd}}^{c}\\ {\hat{U}}_{\mathrm{vq}}^{c} \end{array}\right]\\ +\underset{G_{\mathrm{Hei}}}{\underset{︸}{k_{Q}\left[\begin{array}{cc} U_{\mathrm{Hvq}}^{c} & -U_{\mathrm{Hvd}}^{c} \end{array}\right]}}\left[\begin{array}{c} {\hat{I}}_{d}^{c}\\ {\hat{I}}_{q}^{c} \end{array}\right]) \end{array}$$

Therefore, the HSS representation for generating the voltage reference is given by:

(13) $$\left[\begin{array}{c} {\hat{U}}_{\mathrm{vdref}}^{c}\\ {\hat{U}}_{\mathrm{vqref}}^{c} \end{array}\right]=\underset{G_{\mathrm{Hec}}}{\underset{︸}{\left[\begin{array}{cc} G_{\mathrm{PIec}} & 0\\ 0 & G_{\mathrm{PIec}} \end{array}\right]}}\cdot \left[\begin{array}{c} I_{h}\\ 0 \end{array}\right]\cdot \left(G_{\mathrm{Heuv}}\left[\begin{array}{c} {\hat{U}}_{\mathrm{vd}}^{c}\\ {\hat{U}}_{\mathrm{vq}}^{c} \end{array}\right]+G_{\mathrm{Hei}}\left[\begin{array}{c} {\hat{I}}_{d}^{c}\\ {\hat{I}}_{q}^{c} \end{array}\right]\right)$$

Further incorporating the dynamics of the dual-loop voltage and current control as well as the CCSC, the expressions for **D**_H1_, **E**_H1_ and **D**_H2_, **E**_H2_ can be written as:

(14) $$\left\{\begin{array}{l} D_{H1}=\left[\begin{array}{ccc} I & 0 & 0 \end{array}\right]\left(G_{\mathrm{Hcd}}^{m-1}\left(G_{m}^{i}\left(G_{\mathrm{Hcd}}^{i}+G_{\mathrm{HPLLi}}^{i}\right)+G_{m}^{\mathrm{uv}}G_{\mathrm{HPLLi}}^{\mathrm{uv}}\right)+G_{\mathrm{HPLLi}}^{m-1}\right)S_{i}\\ E_{H1}=\left[\begin{array}{ccc} I & 0 & 0 \end{array}\right]\left(G_{\mathrm{Hcd}}^{m-1}\left(G_{m}^{i}G_{\mathrm{HPLLuv}}^{i}+G_{m}^{\mathrm{uv}}\left(G_{\mathrm{Hcd}}^{\mathrm{uv}}+G_{\mathrm{HPLLuv}}^{\mathrm{uv}}\right)\right)+G_{\mathrm{HPLLuv}}^{m-1}\right)S_{\mathrm{uv}} \end{array}\right.$$

(15) $$\left\{\begin{array}{l} D_{H2}=\left[\begin{array}{ccc} I & 0 & 0 \end{array}\right]G_{\mathrm{Hcd}}^{m-2}\left(G_{\mathrm{Hccsc}2}-G_{\mathrm{Hccsc}1}\right)G_{\mathrm{Hcd}}^{\mathrm{idiff}}S_{\mathrm{idiff}}\\ E_{H2}=\left[\begin{array}{ccc} I & 0 & 0 \end{array}\right]\left(G_{\mathrm{Hcd}}^{m2-1}\left(G_{\mathrm{Hccsc}2}-G_{\mathrm{Hccsc}1}\right)G_{\mathrm{HPLLuv}}^{\mathrm{idiff}}+G_{\mathrm{HPLLuv}}^{m2-1}\right)S_{\mathrm{uv}}\\ F_{H2}=\left[\begin{array}{ccc} I & 0 & 0 \end{array}\right]\left(G_{\mathrm{Hcd}}^{m2-1}\left(G_{\mathrm{Hccsc}2}-G_{\mathrm{Hccsc}1}\right)G_{\mathrm{HPLLi}}^{\mathrm{idiff}}+G_{\mathrm{HPLLi}}^{m2-1}\right)S_{i} \end{array}\right.$$

Subsequently, the impedance model of the VSG-controlled WFMMC can be obtained using Equations (6) and (7).

For the V-f controlled MMC, since its phase angle is derived by directly integrating a constant, no harmonic coupling elements exist. The expression for $\hat{\theta }$ can be expressed as:

(16) $$\hat{\theta }={\left[\begin{array}{ccc} \begin{array}{ccc} 0 & \cdots & 0 \end{array} & \omega_{0}/s & \begin{array}{ccc} 0 & \cdots & 0 \end{array} \end{array}\right]}^{T}$$

Consequently, in Equations (14) and (15), all matrices containing phase angle dynamics are set to zero. These specifically include:

(17) $$\begin{array}{l} G_{\mathrm{HPLLuv}}^{\mathrm{uv}},\;G_{\mathrm{HPLLi}}^{\mathrm{uv}},\;G_{\mathrm{HPLLuv}}^{i},\;G_{\mathrm{HPLLi}}^{i},\;G_{\mathrm{HPLLi}}^{m-1},\;G_{\mathrm{HPLLuv}}^{m-1}=0\\ G_{\mathrm{HPLLuv}}^{\mathrm{idiff}},\;G_{\mathrm{HPLLi}}^{\mathrm{idiff}},\;G_{\mathrm{HPLLuv}}^{m2-1},\;G_{\mathrm{HPLLi}}^{m2-1}=0 \end{array}$$

Furthermore, since the d-axis voltage reference for the V-f-controlled WFMMC is set directly to the rated AC voltage, Equation (13) simplifies to:

(18) $$\left[\begin{array}{c} {\hat{U}}_{\mathrm{vdref}}^{c}\\ {\hat{U}}_{\mathrm{vqref}}^{c} \end{array}\right]=\cdot \left[\begin{array}{c} I_{h}\\ 0 \end{array}\right]$$

Finally, under V-f control, Equations (14) and (15) reduce to:

(19) $$\left\{\begin{array}{l} D_{H1}=\left[\begin{array}{ccc} I & 0 & 0 \end{array}\right]\left(G_{\mathrm{Hcd}}^{m-1}G_{m}^{i}G_{\mathrm{Hcd}}^{i}S_{i}\right)\\ E_{H1}=\left[\begin{array}{ccc} I & 0 & 0 \end{array}\right]\left(G_{\mathrm{Hcd}}^{m-1}G_{m}^{\mathrm{uv}}G_{\mathrm{Hcd}}^{\mathrm{uv}}S_{\mathrm{uv}}\right) \end{array}\right.$$

(20) $$\left\{\begin{array}{l} D_{H2}=\left[\begin{array}{ccc} I & 0 & 0 \end{array}\right]G_{\mathrm{Hcd}}^{m-2}\left(G_{\mathrm{Hccsc}2}-G_{\mathrm{Hccsc}1}\right)G_{\mathrm{Hcd}}^{\mathrm{idiff}}S_{\mathrm{idiff}}\\ E_{H2}=0\\ F_{H2}=0 \end{array}\right.$$

Subsequently, the impedance model of the V-f-controlled WFMMC can be derived using Equations (6) and (7).

Based on the impedance matrix **Z**_pnMMC_ of the WFMMC, the relationship between the positive- and negative-sequence voltages and currents at its output port can be expressed as:

(21) $$\left[\begin{array}{c} \Delta u_{\mathrm{MMC}}\left(s+j\omega_{0}\right)\\ \Delta u_{\mathrm{MMC}}\left(s-j\omega_{0}\right) \end{array}\right]=\underset{Z_{\mathrm{pnMMC}}}{\underset{︸}{\left[\begin{array}{cc} Z_{\mathrm{pp}} & Z_{\mathrm{pn}}\\ Z_{\mathrm{np}} & Z_{\mathrm{nn}} \end{array}\right]}}\left[\begin{array}{c} \Delta i_{\mathrm{MMC}}\left(s+j\omega_{0}\right)\\ \Delta i_{\mathrm{MMC}}\left(s-j\omega_{0}\right) \end{array}\right]$$

**Figure 4:**
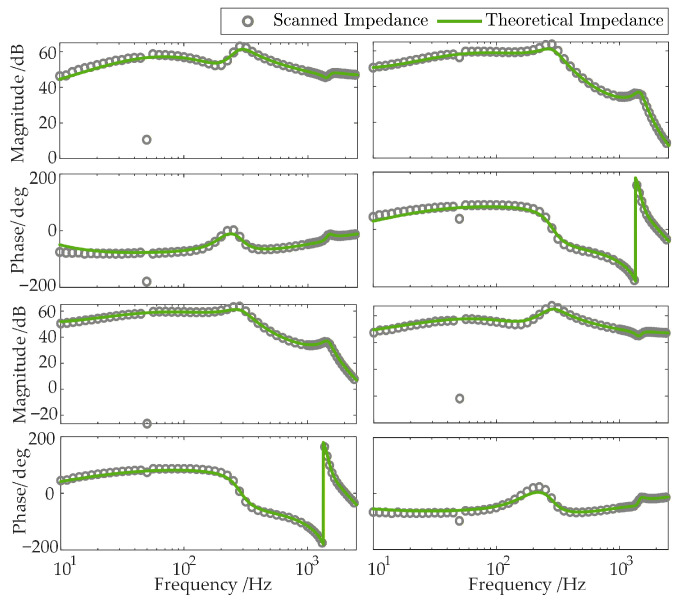
Impedance model verification results for the GSC.

**Figure 5:**
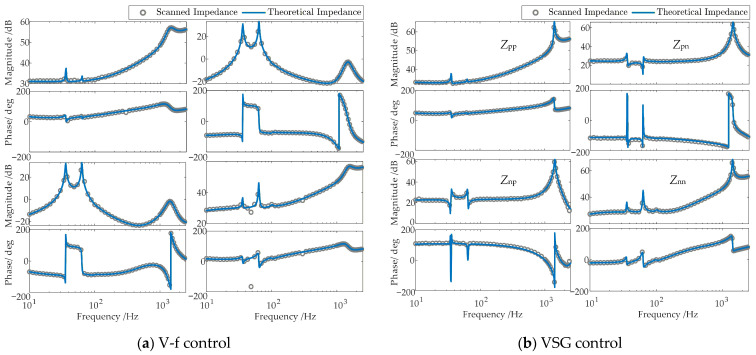
Impedance model verification results for the WFMMC.

To verify the accuracy of the aforementioned impedance models, a time-domain electromagnetic transient (EMT) simulation model of the MMC-HVDC-integrated offshore wind farms, as depicted in Figure 1, is established in MATLAB/Simulink (R2023a). The model’s specific electrical and control parameters are fully consistent with those listed in Appendix A Table A1, Table A2 and Table A3. The impedances of the GSC, the V-f-controlled WFMMC, and the VSG-controlled WFMMC are measured separately in the time-domain EMT simulation using the frequency-scanning method. The comparative results between these theoretical impedance models and the frequency-scanning impedances are presented in Figure 4 and Figure 5, respectively.

As shown in the figures, the theoretical impedance models closely match the frequency-scanning impedances. Therefore, these models are suitable for theoretical analysis of high-frequency oscillations and frequency coupling in the system.

### 2.3. Conventional High-Frequency Stability Analysis Without Considering Frequency Coupling

Under the premise of neglecting the high-frequency-range frequency coupling between the GSC and the WFMMC [26], the system stability is assessed by calculating the phase margin from the positive-sequence impedance *Z*_ppPMSG_ of the GSC’s impedance matrix **Z**_pnPMSG_ and the positive-sequence impedance *Z*_ppMMC_ of the WFMMC’s impedance matrix **Z**_pnMMC_ [37]. Specifically, at the frequency where the magnitudes of *Z*_ppPMSG_ and *Z*_ppMMC_ are equal, the system is considered stable if the phase difference is less than or equal to 180°; otherwise, it is unstable.

**Figure 6:**
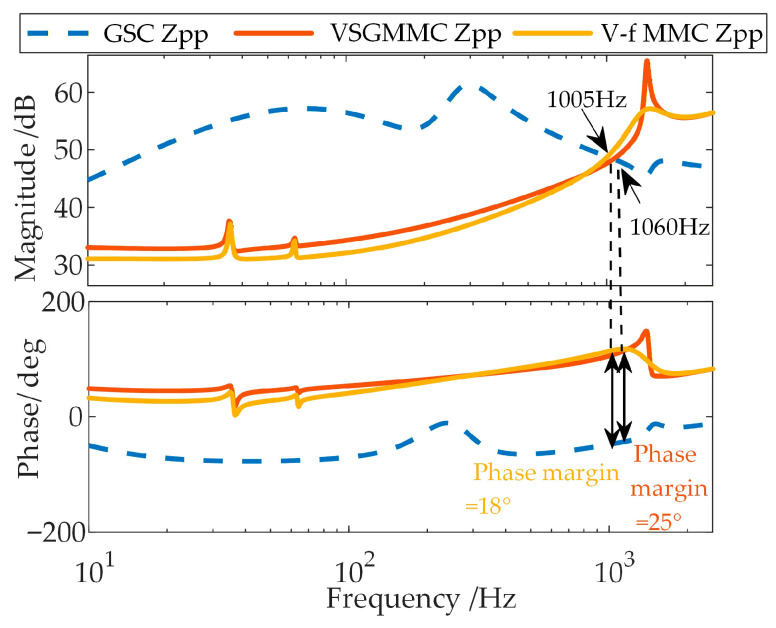
Stability analysis results of the system.

Applying this analysis method to assess the stability between the GSC and the WFMMC under the two control strategies yields the results shown in Figure 6.

As shown in Figure 6, when high-frequency-range frequency coupling is neglected, the phase margins at the frequency where the magnitudes of the positive-sequence impedances of the GSC and the WFMMC under the two control strategies are equal are 18° and 25°, respectively, both of which are positive. Therefore, under the electrical and control parameters specified in Appendix A Table A1, Table A2 and Table A3, the system should remain stable according to this analysis.

**Figure 7:**
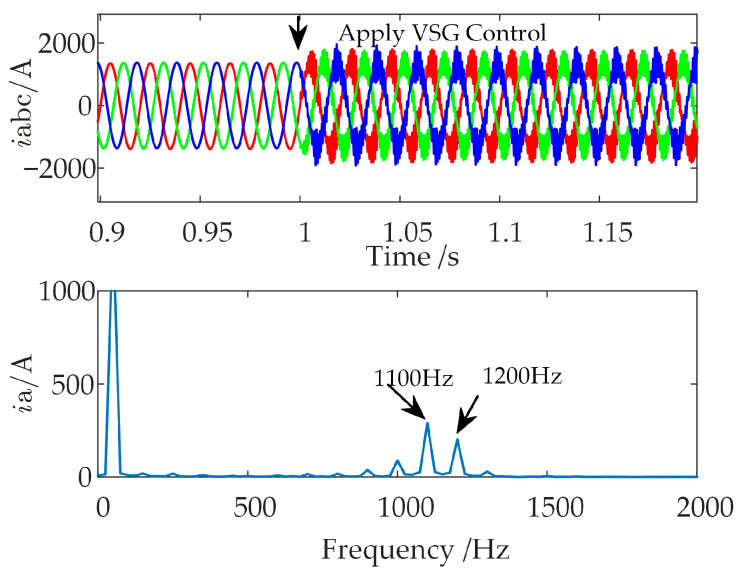
Time-domain simulation results before and after switching WFMMC to VSG control.

In the time-domain EMT simulation model established in Section 2.2, the WFMMC initially operates under V-f control. Keeping all other conditions unchanged, the virtual synchronous and virtual excitation loops are activated at *t* = 1 s, switching the WFMMC’s control strategy to VSG control. The resulting waveform of the WFMMC output current and the Fast Fourier Transform (FFT) analysis for the interval from 1.02 s to 1.06 s are presented in Figure 7.

As shown in Figure 7, the system remains stable before *t* = 1 s when the WFMMC operates under V-f control. However, after the WFMMC switches to VSG control, high-frequency oscillations with frequencies of 1100 Hz and 1200 Hz emerge. This discrepancy between the time-domain simulation results and the theoretical stability analysis indicates that the conventional high-frequency stability analysis method, which considers only the positive-sequence impedances of the GSC and the WFMMC while neglecting frequency coupling, is inadequate when the WFMMC employs VSG control.

**Figure 8:**
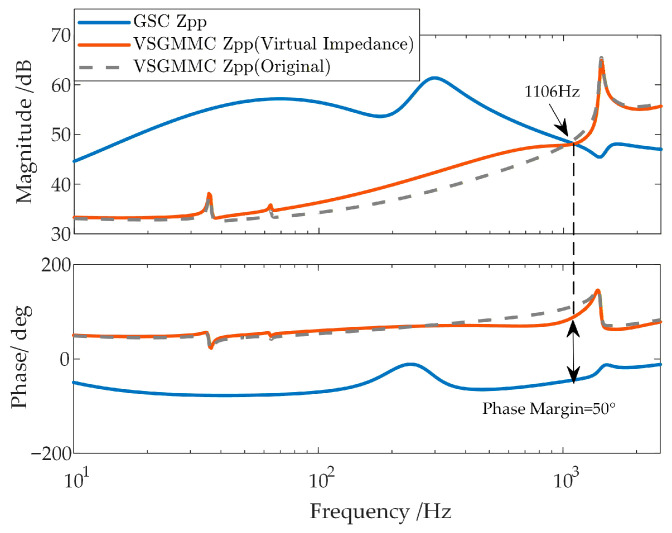
Modification of the WFMMC impedance characteristics by virtual impedance.

The high-frequency oscillation is suppressed using the virtual impedance strategy proposed in Reference [30]. The stability analysis results of the system before and after suppression are shown in Figure 8.

**Figure 9:**
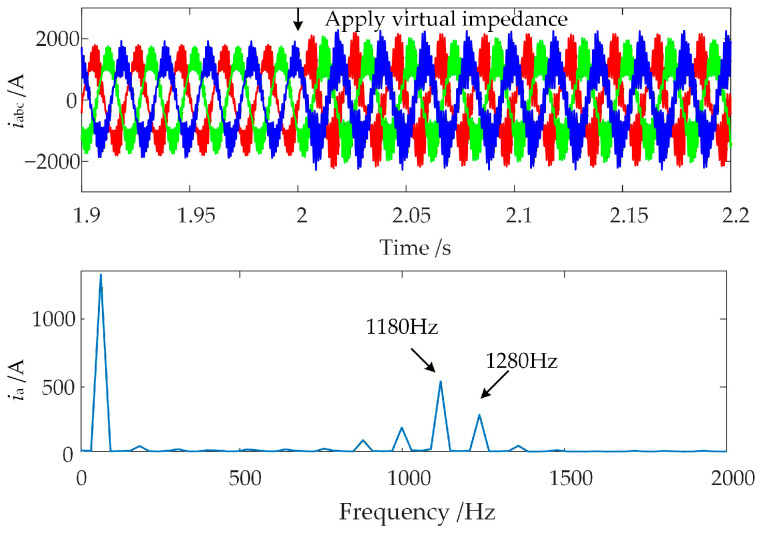
Time-domain simulation results after applying virtual impedance.

It can be observed that the virtual impedance suppression strategy effectively mitigates the high-frequency inductive negative damping of the WFMMC and improves the phase margin to 50°, seemingly demonstrating a good oscillation suppression effect. However, the time-domain simulation results after applying the virtual impedance suppression strategy are shown in Figure 9.

After applying the virtual impedance suppression strategy at *t* = 2 s, the high-frequency oscillation phenomenon in the system remains unsuppressed. Therefore, it is necessary to further consider the frequency coupling effects between the GSC and the VSG-controlled WFMMC in the high-frequency range and design a new high-frequency oscillation suppression strategy based on the frequency coupling mechanism.

## 3. Analysis of Frequency Coupling and Influencing Factors

### 3.1. Mechanism Analysis of Frequency Coupling Between GSC and WFMMC

The frequency coupling effect between the GSC and the WFMMC influences their respective positive-/negative-sequence disturbance-to-response transfer functions. To more intuitively analyze the impact of the frequency coupling effect on the impedances of the GSC and the WFMMC separately, this article utilizes signal flow graphs based on the GSC’s admittance matrix **Y**_pnPMSG_ and the WFMMC’s impedance matrix **Z**_pnMMC_, analyzing the frequency coupling process from both the GSC’s perspective and the WFMMC’s perspective.

**Figure 10:**
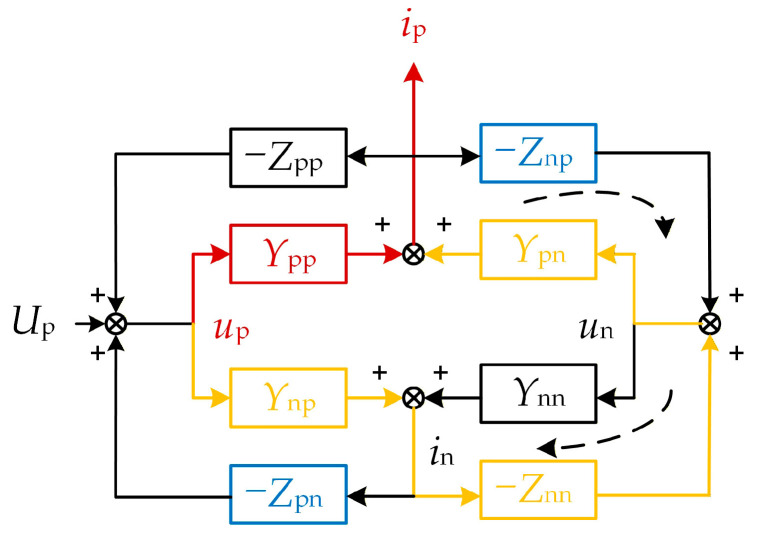
Frequency coupling mechanism from the GSC’s perspective.

The frequency coupling process from the GSC’s perspective is illustrated in Figure 10. For the GSC, when frequency coupling is neglected, the elements of the WFMMC impedance matrix representing its influence (*Z*_pp_, *Z*_pn_, *Z*_np_, and *Z*_nn_) are all considered zero. When a positive-sequence voltage disturbance *u*_p_ is injected at the PCC, according to the positive-/negative-sequence voltage/current relationship shown in Equation (4) (where (*s* + j*ω*_0_) denotes the positive sequence, p, and (*s* − j*ω*_0_) denotes the negative sequence, n), the forward path from the disturbance *u*_p_ to the positive-sequence current response *i*_p_ is solely the red path indicated in Figure 10. The positive-sequence admittance in this case is simply *Y*_pp_.

When the frequency coupling effect is considered, the positive-sequence voltage disturbance *u*_p_ additionally generates an extra positive-sequence current response through the forward path formed jointly by the GSC’s admittance and the WFMMC’s impedance, as shown by the yellow path in Figure 10. Furthermore, the entire system incorporates two additional control loops, also formed jointly by the GSC’s admittance and the WFMMC’s impedance, indicated by the black dashed lines in Figure 10.

Applying Mason’s formula, the transfer function from the positive-sequence current response *i*_p_ to the positive-sequence voltage disturbance *u*_p_, which is the equivalent positive-sequence impedance of the GSC, can be derived as:

(22) $$Z_{\mathrm{SISO}}^{\mathrm{PMSG}}=\frac{u_{p}}{i_{p}}={\left\{Y_{\mathrm{pp}}-\frac{Y_{\mathrm{pp}}Z_{\mathrm{np}}Y_{\mathrm{pn}}+Y_{\mathrm{np}}Z_{\mathrm{nn}}Y_{\mathrm{pn}}}{1+Z_{\mathrm{nn}}Y_{\mathrm{nn}}+Z_{\mathrm{np}}Y_{\mathrm{pn}}}\right\}}^{-1}$$

It is evident that, after accounting for the frequency coupling effect, the positive-sequence impedance of the GSC is modified, incorporating the additional term presented in Equation (22).

**Figure 11:**
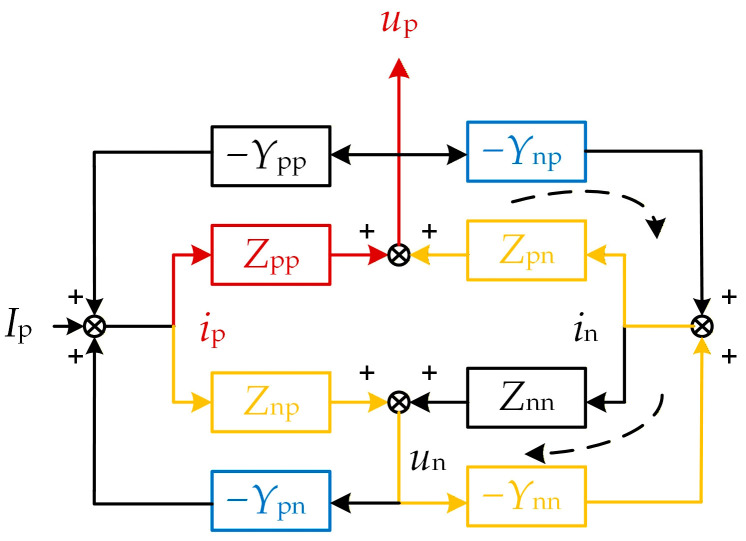
Frequency coupling mechanism from the WFMMC’s perspective.

The frequency coupling process from the WFMMC’s perspective is illustrated in Figure 11. For the WFMMC, when frequency coupling is neglected, the elements of the GSC admittance matrix representing its influence (*Y*_pp_, *Y*_pn_, *Y*_np_, *Y*_nn_) are all considered zero. When a positive-sequence current disturbance *i*_p_ is injected at the PCC, according to the positive-/negative-sequence voltage/current relationship shown in Equation (21), the forward path from the disturbance *i*_p_ to the positive-sequence voltage response *u*_p_ is solely the red path indicated in Figure 11. The positive-sequence impedance in this case is simply *Z*_pp_.

When the frequency coupling effect is considered, the positive-sequence current disturbance *i*_p_ additionally generates an extra positive-sequence voltage response through the forward path formed jointly by the GSC’s admittance and the WFMMC’s impedance, as shown by the yellow path in Figure 11. Furthermore, the entire system incorporates two additional control loops, also formed jointly by the GSC’s admittance and the WFMMC’s impedance, indicated by the black dashed lines in Figure 11.

Applying Mason’s formula, the transfer function from the positive-sequence current disturbance *i*_p_ to the positive-sequence voltage response *u*_p_, which is the equivalent positive-sequence impedance of the WFMMC, can be derived as:

(23) $$Z_{\mathrm{SISO}}^{\mathrm{MMC}}=Z_{\mathrm{pp}}-\frac{Z_{\mathrm{pp}}Y_{\mathrm{np}}Z_{\mathrm{pn}}+Z_{\mathrm{pn}}Y_{\mathrm{nn}}Z_{\mathrm{np}}}{1+Z_{\mathrm{nn}}Y_{\mathrm{nn}}+Z_{\mathrm{np}}Y_{\mathrm{pn}}}$$

When the frequency coupling effect is accounted for, the positive-sequence impedance of the WFMMC is also modified, incorporating the additional term presented in Equation (23).

**Figure 12:**
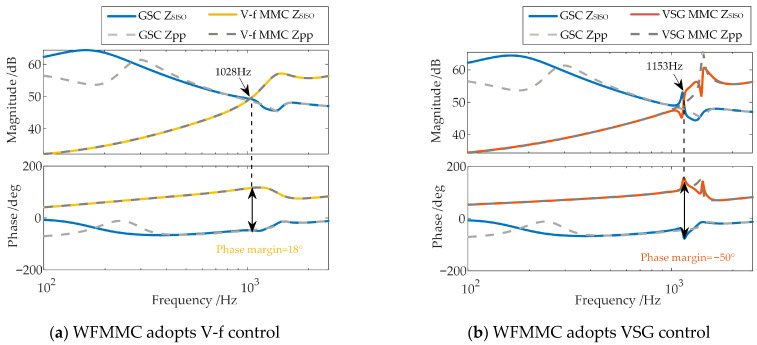
Stability analysis for the system, with and without considering frequency coupling.

The stability of the scenario described in Section 2.3 is re-analyzed using the equivalent positive-sequence impedances that consider frequency coupling, with the results shown in Figure 12. It can be observed that within the high-frequency range around 1000 Hz, where an oscillation risk exists, the frequency coupling effect has a certain impact on the GSC’s positive-sequence impedance when the WFMMC employs V-f control, but its influence on the WFMMC’s positive-sequence impedance is minimal. The system’s phase margin remains at 18°, showing almost no change compared to the analysis neglecting coupling. However, after the WFMMC switches to VSG control, the frequency coupling effect severely impacts the positive-sequence impedances of both the GSC and the WFMMC within the risk-prone high-frequency range. The system’s phase margin deteriorates to −50°. The oscillation risk frequency identified in the modified harmonic domain, based on the established impedance models, is 1153 Hz (corresponding to 1103 Hz and 1203 Hz in the time domain), which aligns with the FFT analysis results shown in Figure 7.

Therefore, unlike the scenario where the WFMMC employs V-f control, the frequency coupling effect between the GSC and the VSG-controlled WFMMC is not confined to the sub-/super-synchronous frequency range. A significant frequency coupling effect also exists in the high-frequency range influenced by control delay. This high-frequency-range frequency coupling is precisely what triggers the high-frequency oscillation between the GSC and the VSG-controlled WFMMC observed in Section 2.3.

### 3.2. Analysis of Key Influencing Factors for Frequency Coupling

To quantitatively analyze the influence of control loops and parameters on the frequency coupling effect within the system, this article defines a frequency coupling intensity indicator, as shown in Equation (24), based on the degree to which frequency coupling modifies the converter’s impedance. The numerator of Equation (24) represents the change in the positive-sequence impedance induced by frequency coupling. Thus, a larger value of this indicator indicates a greater impact of frequency coupling on the impedance of either the GSC or the WFMMC, corresponding to stronger frequency coupling.

(24) $$\xi =\frac{\left|Z_{\mathrm{SISO}}-Z_{\mathrm{pp}}\right|}{\left|Z_{\mathrm{pp}}\right|}$$

As observed in Figure 12, after the activation of the virtual synchronous and virtual excitation loops in the WFMMC’s VSG control, the frequency coupling phenomenon undergoes a significant change compared to the V-f control case. Furthermore, Figure 12 indicates that high-frequency-range frequency coupling primarily occurs at the high-frequency resonant peaks in the WFMMC’s impedance characteristics. The emergence of such peaks is attributed to the control delay of the WFMMC [34]. Therefore, this article employs the frequency coupling intensity indicator defined in Equation (24) to analyze the influence of the VSG’s virtual synchronous and virtual excitation loops, as well as the WFMMC’s control delay, on the frequency coupling.

**Figure 13:**
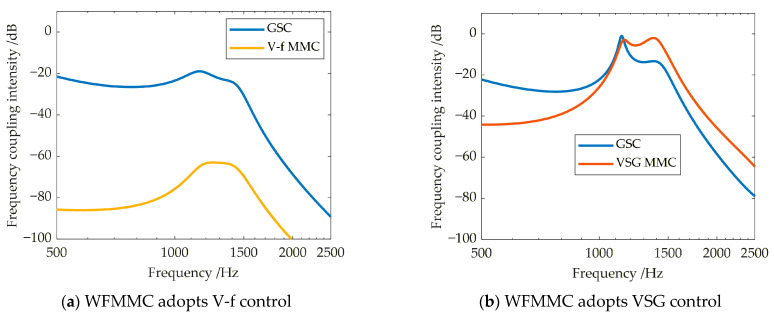
Frequency coupling intensity in the system with WFMMC under two control strategies.

Focusing on the active support components (virtual synchronous and virtual excitation) added by the VSG control, the high-frequency-range frequency coupling intensity between the GSC and the WFMMC is compared before and after their activation, while keeping all other parameters constant. The results are shown in Figure 13a,b, with the maximum values of their respective frequency coupling intensity indicators within the 500–2500 Hz high-frequency range presented in Table 1.

It is evident that compared to the case where the WFMMC employs V-f control, the activation of the additional virtual synchronous and virtual excitation control loops in the WFMMC leads to a significant increase in the frequency coupling intensity between the GSC and the WFMMC within the system. Consequently, the equivalent positive-sequence impedances of both converters are subject to modification from frequency coupling.

**Figure 14:**
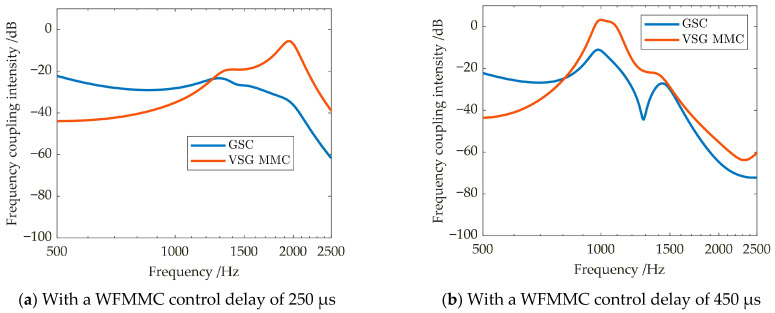
Frequency coupling intensity in the system under different WFMMC control delays.

Due to its numerous submodules, the WFMMC exhibits a relatively large control delay, which is composed of sampling delay, central control delay, and internal communication delay [38]. Regarding the impact of the WFMMC’s control delay on frequency coupling, under the condition that the WFMMC operates with VSG control and all other parameters remain constant, the high-frequency-range frequency coupling intensity between the GSC and the WFMMC is compared for different WFMMC control delays. The results are shown in Figure 14a,b, with the maximum values of their respective frequency coupling intensity indicators within the 500–2500 Hz high-frequency range presented in Table 2.

It is evident that when the WFMMC employs VSG control, increasing its control delay from 250 µs to 450 µs results in a significant rise in the frequency coupling intensity between the GSC and the WFMMC within the system. Consequently, the equivalent positive-sequence impedances of both converters undergo substantial modifications due to this intensified frequency coupling.

Based on the above analysis, the virtual synchronous and virtual excitation loops within the WFMMC’s VSG control, along with the WFMMC’s control delay, are identified as the key factors influencing the high-frequency-range frequency coupling effect. The activation of VSG control and an increase in control delay intensify this coupling in the high-frequency range, thereby exerting a destabilizing effect on the system. This phenomenon can be further explained by referring to the control block diagram in Figure 3. Compared to the symmetric control structure under V-f control, the introduction of the virtual synchronous and virtual excitation loops in VSG control significantly heightens the control asymmetry of the WFMMC. Simultaneously, the WFMMC’s control delay, acting through processes such as voltage/current sampling and power calculation, interacts with these asymmetric control loops. This interaction amplifies the magnitudes of the off-diagonal elements in the impedance matrices of both the WFMMC and the GSC within the high-frequency range, leading to a marked enhancement of the frequency coupling effect in this frequency band. This conclusion provides valuable guidance for the subsequent design of high-frequency oscillation suppression strategies.

## 4. High-Frequency Oscillation Suppression Strategy Considering Frequency Coupling

### 4.1. Oscillation Suppression Strategy Based on Band-Stop Filtering in WFMMC Voltage Sampling

**Figure 15:**
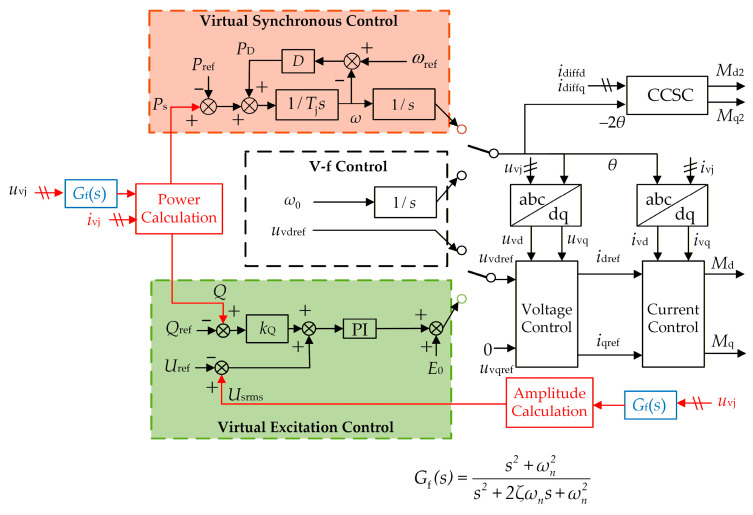
Key control paths influencing high-frequency-range frequency coupling and control structure of the suppression strategy.

To effectively suppress the high-frequency oscillations induced by frequency coupling between the GSC and the VSG-controlled WFMMC, it is essential to design a targeted suppression strategy based on the key control loops that influence high-frequency-range frequency coupling. According to the theoretical analysis in Section 3.2, these critical control loops are represented by the red paths illustrated in Figure 15.

It is evident that the key control paths influencing high-frequency-range frequency coupling primarily include voltage/current sampling, power calculation, and voltage magnitude calculation. To simplify the control structure of the oscillation suppression strategy as much as possible, this article opts to implement an additional filter within the voltage sampling loop—a common component across the aforementioned paths—to design the high-frequency oscillation suppression strategy. Its control structure is illustrated by the blue section in Figure 15.

Given that the high-frequency-range frequency coupling effect is significantly influenced by the WFMMC’s control delay and primarily manifests in the frequency ranges where this delay causes resonant peaks in the impedance characteristics, a second-order band-stop filter is adopted as the additional filtering in the proposed strategy. This choice aims to effectively impede the impact of WFMMC delay on frequency coupling in these critical frequency ranges while minimizing adverse effects on the system’s dynamic performance. The transfer function of this filter is:

(25) $$G_{f}=\frac{s^{2}+{\left(2\pi f_{n}\right)}^{2}}{s^{2}+2\zeta \left(2\pi f_{n}\right)s+{\left(2\pi f_{n}\right)}^{2}}$$

where ζ is the damping ratio of the band-stop filter, typically set to 0.5, and *f*_n_ is its center frequency.

When designing the center frequency of the band-stop filter, it should be placed near the frequency where the control delay exerts the most significant influence on the WFMMC’s impedance characteristics to optimally block the path through which the control delay exacerbates frequency coupling. Based on the authors’ previous work [24] and the results shown in Figure 5, a control delay *T*_d_ causes a high-frequency resonant peak to appear in the WFMMC’s impedance characteristics near (0.5/*T*_d_) Hz, around which the elements of the WFMMC’s impedance matrix undergo drastic changes. Therefore, the center frequency of the band-stop filter in proposed strategy is selected as:

(26) $$f_{n}=\frac{1}{2T_{d}}$$

### 4.2. Effectiveness of the Proposed Strategy in Mitigating Frequency Coupling and High-Frequency Oscillations

**Figure 16:**
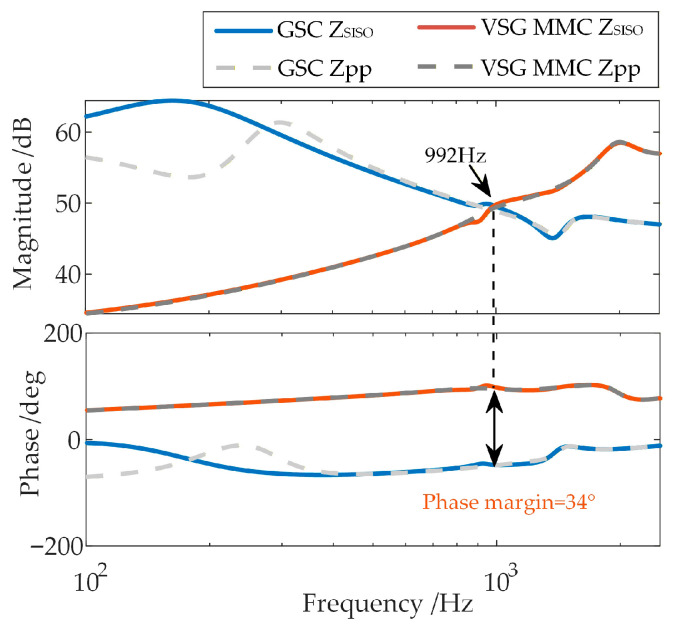
Stability analysis after applying the proposed strategy.

After applying the proposed oscillation suppression strategy, the stability of the GSC and WFMMC is re-analyzed using their equivalent impedances that account for frequency coupling, based on the high-frequency oscillation scenario from Section 2.3. In this case, the WFMMC control delay is 350 µs, and the center frequency of the band-stop filter is set to 1429 Hz. The stability analysis results are shown in Figure 16.

It can be observed that, with the proposed strategy applied, the equivalent positive-sequence impedances (*Z*_SISO_) of both converters in the high-frequency range show little difference from their original positive-sequence impedances (*Z*_pp_), indicating a weakened frequency coupling effect. The system phase margin, derived from these equivalent impedances *Z*_SISO_, improves to 34°. Consequently, the system can maintain stability under the scenario described in Section 2.3.

**Figure 17:**
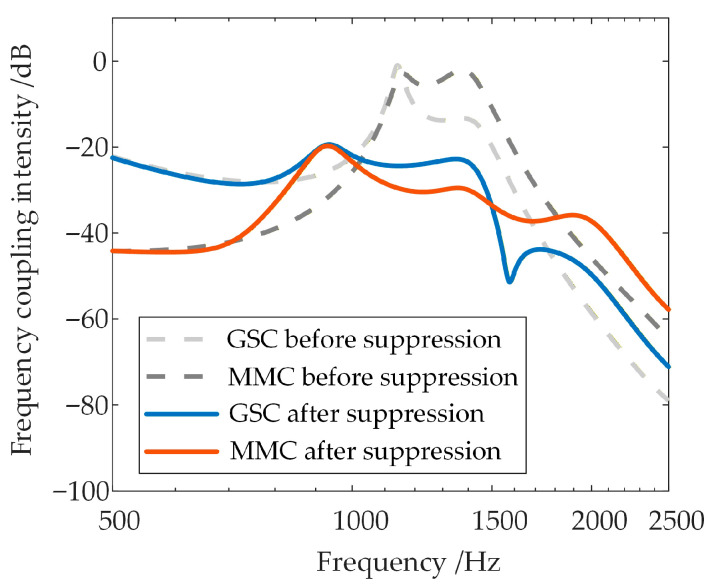
Comparison of frequency coupling intensity before and after applying the proposed strategy.

A comparative analysis of the system’s frequency coupling intensity before and after applying the proposed strategy is conducted using the frequency coupling intensity indicator. The results, shown in Figure 17 and Table 3, demonstrate a significant reduction in the frequency coupling intensity indicator within the risk-prone frequency ranges after the application of the proposed strategy.

It is evident that the proposed high-frequency oscillation suppression strategy effectively weakens the frequency coupling effect between the GSC and the VSG-controlled MMC in the high-frequency range, thereby suppressing the oscillations induced by this coupling.

**Figure 18:**
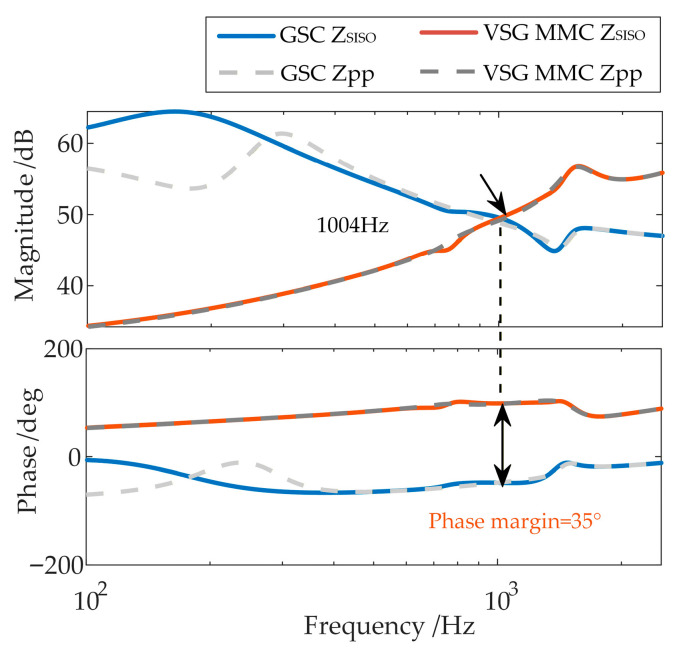
Stability analysis after applying the proposed strategy under severe delay conditions.

The effectiveness of the proposed strategy in mitigating frequency coupling and high-frequency oscillations is further analyzed under more severe WFMMC delay conditions. After the WFMMC control delay is increased to 450 µs, the center frequency of the band-stop filter is set to 1111 Hz. The stability of the GSC and the WFMMC is then analyzed using their equivalent impedances that account for frequency coupling after the application of the proposed strategy. The results are presented in Figure 18.

In this case, the frequency coupling effect between the two converters in the high-frequency range remains similarly weak. The system phase margin, derived from the equivalent positive-sequence impedances (*Z*_SISO_) of the GSC and WFMMC, is 35°, indicating that the system can still maintain stability.

**Figure 19:**
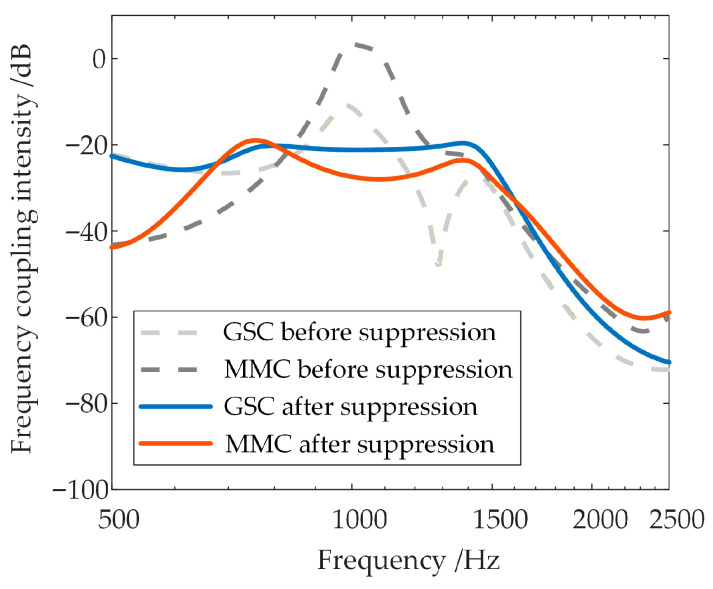
Comparison of frequency coupling intensity before and after applying the proposed strategy with delay increased to 450 µs.

Using the frequency coupling intensity indicator, a comparative analysis of the system’s coupling intensity before and after applying the proposed suppression strategy is conducted after the control delay is increased to 450 µs. The results are shown in Figure 19 and Table 4.

It is evident that even with the control delay increased to 450 µs, the frequency coupling intensity indicator within the risk-prone ranges shows a significant reduction after the application of the proposed strategy.

In summary, because the proposed high-frequency oscillation suppression strategy is designed based on the key path influenced by control delay, it exhibits strong robustness against increases in delay. Even under larger delays, the strategy remains effective in weakening the high-frequency-range coupling effect between the GSC and the VSG-controlled WFMMC and in suppressing the resulting oscillations.

### 4.3. Comparison of the Effectiveness Between the Proposed Strategy and the Conventional Virtual Impedance Strategy

**Figure 20:**
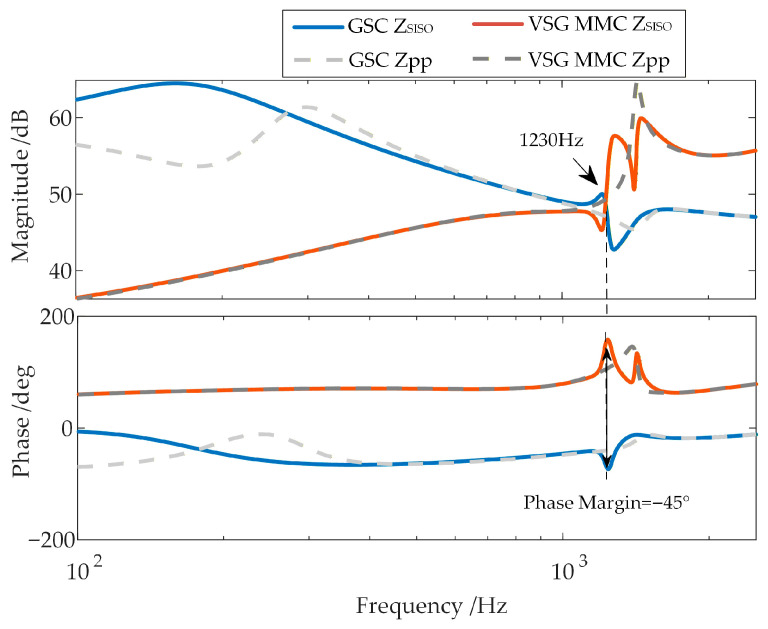
Stability analysis after applying the virtual impedance strategy.

Furthermore, this article compares the oscillation suppression performance of the proposed strategy with that of the virtual impedance strategy in Reference [30]. Stability analysis is conducted using the equivalent positive-sequence impedance considering frequency coupling, and the results are shown in Figure 20.

**Figure 21:**
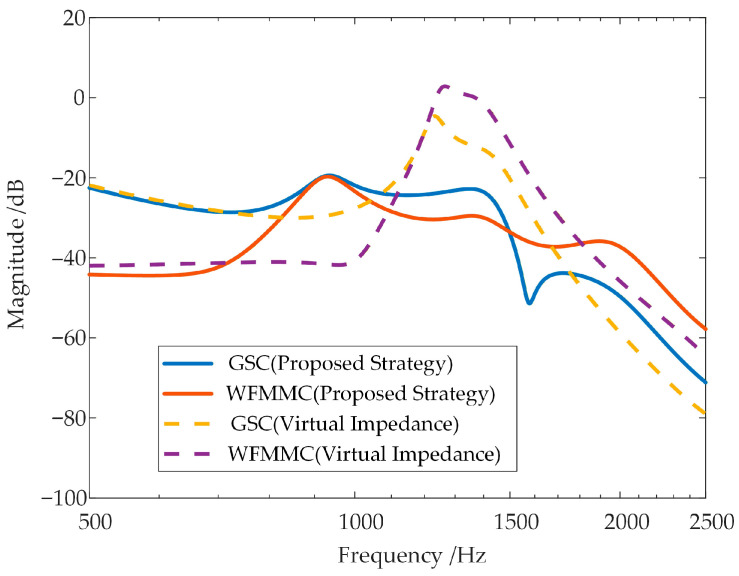
Comparison of frequency coupling intensity between the proposed strategy and virtual impedance.

As shown in the figure, although the virtual impedance can reduce the inductive negative damping of the VSG-controlled WFMMC itself, the frequency coupling effect between the GSC and the VSG-controlled WFMMC is not weakened. A significant frequency coupling still exists between them in the high-frequency band, leading to a deterioration of the system phase margin and inducing high-frequency oscillations. This is also confirmed by the frequency coupling intensity indicator between the GSC and the VSG-controlled WFMMC shown in Figure 21.

Finally, this article provides a quantitative summary of the frequency coupling mitigation and oscillation suppression performance of the proposed voltage sampling filtering strategy and the conventional virtual impedance strategy through a summary table, as shown in Table 5.

According to Table 5, compared with the conventional virtual impedance strategy, the proposed strategy can significantly weaken the frequency coupling between the GSC and the WFMMC, thereby achieving a higher phase margin.

## 5. Simulation Verification

Based on MATLAB/Simulink, the effectiveness of the proposed high-frequency oscillation suppression strategy is verified within the time-domain EMT simulation model of the MMC-HVDC-integrated offshore wind farms established in Section 2.2.

**Figure 22:**
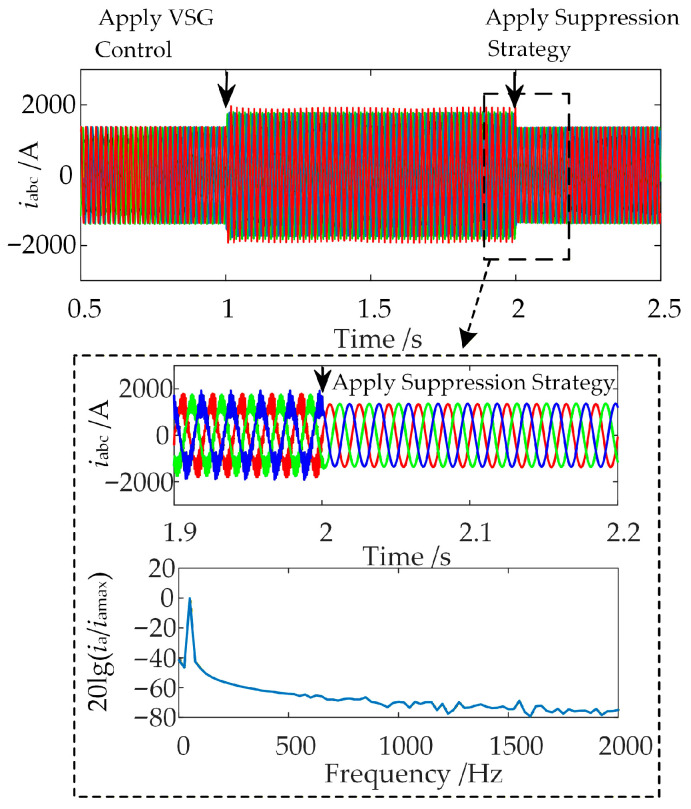
Time-domain simulation results after applying the proposed strategy.

Focusing on the high-frequency oscillation phenomenon observed in Section 2.3 between the GSC and the VSG-controlled WFMMC, the simulation is configured as follows: the WFMMC initially operates under V-f control. At *t* = 1 s, the virtual synchronous and virtual excitation loops are activated, switching the WFMMC’s control to VSG mode, which triggers high-frequency oscillations in the system. Subsequently, at *t* = 2 s, the proposed high-frequency oscillation suppression strategy is applied, with the band-stop filter’s center frequency set to 1429 Hz. The resulting waveform of the WFMMC output current and the FFT analysis for the interval from 2.02 s to 2.06 s are presented in Figure 22.

As shown in Figure 22, after the proposed high-frequency oscillation suppression strategy is applied at *t* = 2 s, the high-frequency oscillations in the system are rapidly eliminated. The FFT results indicate that the output current now contains predominantly the fundamental frequency component, confirming the system’s return to stability. This demonstrates that the proposed suppression strategy considering frequency coupling is effective in mitigating the high-frequency oscillations induced by frequency coupling between the GSC and the VSG-controlled WFMMC.

**Figure 23:**
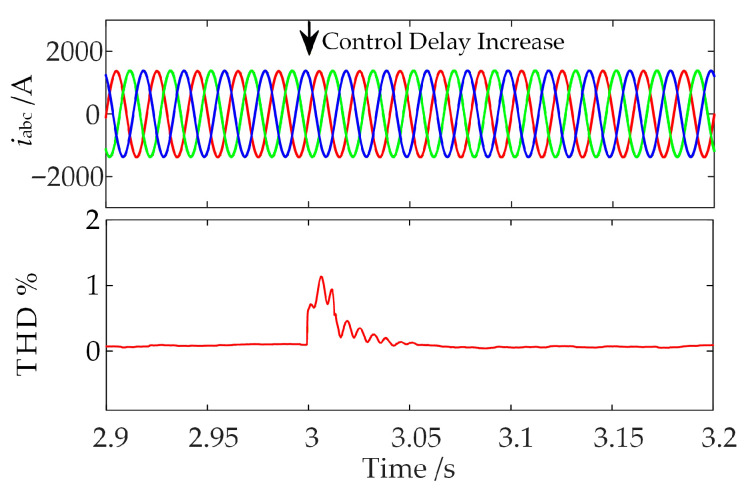
Time-domain simulation verification of the proposed strategy effectiveness under increased delay.

Proceeding further, at *t* = 3 s, the control delay of the WFMMC is increased to 450 µs, and the center frequency of the band-stop filter in the suppression strategy is adjusted to 1111 Hz. The resulting waveform of the WFMMC output current and its Total Harmonic Distortion (THD) are shown in Figure 23.

As shown in Figure 23, under the severe condition of an increased control delay at *t* = 3 s, the WFMMC output current rapidly returns to a steady-state operating point after a brief transient following the application of the proposed high-frequency oscillation suppression strategy. The current’s THD remains consistently low, and the system maintains stability. These simulation results demonstrate that the proposed strategy can mitigate the impact of control delay on frequency coupling, adapt to variations in WFMMC control delay, and exhibits strong robustness.

**Figure 24:**
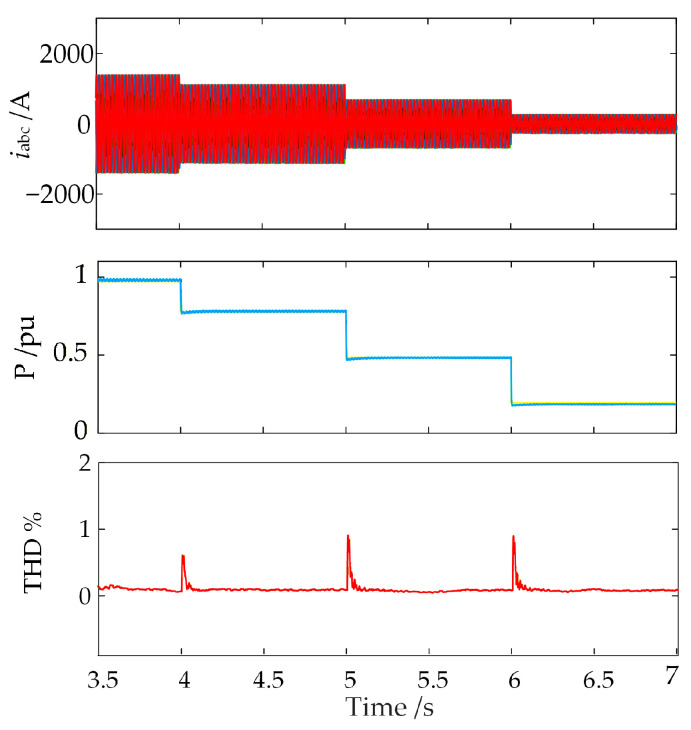
Time-domain simulation verification of the proposed strategy effectiveness under active power reference adjustment.

Furthermore, given that the output power of offshore wind farms fluctuates frequently with weather conditions, it is necessary to verify the effectiveness of the proposed strategy under scenarios of frequent power variations. After setting the WFMMC control delay to 450 µs, the reference values for the output active power of the GSC and the input active power of the WFMMC are reduced from 1.0 pu to 0.8 pu, 0.5 pu, and 0.2 pu at *t* = 4 s, *t* = 5 s, and *t* = 6 s, respectively, within the time-domain simulation model. The resulting AC of the WFMMC, its input active power, and the THD of the AC are presented in Figure 24.

As shown in Figure 24, under complex operating conditions involving frequent active power variations, the WFMMC’s current and active power can rapidly transition to a new steady-state operating point after the application of the proposed high-frequency oscillation suppression strategy. The current’s THD remains consistently low, and the system maintains stability throughout. These simulation results confirm the adaptability and engineering practicality of the proposed oscillation suppression strategy for MMC-HVDC-integrated offshore wind farms under complex operating conditions.

## 6. Conclusions

In this article, a high-frequency oscillation suppression strategy that considers frequency coupling and is based on band-stop filtering in the WFMMC voltage sampling is proposed. The primary findings and conclusions are summarized as follows:

1. Frequency coupling between the GSC and the WFMMC is not confined to the sub-/super-synchronous frequency range. Significant frequency coupling occurs in the high-frequency range when the WFMMC adopts VSG control, which can induce high-frequency oscillations. The asymmetric control structure of the VSG control, along with the WFMMC control delay, are identified as the key factors responsible for the high-frequency-range frequency coupling.
2. Based on the key control path through which control delay act upon the asymmetric control structure to cause high-frequency-range frequency coupling, a high-frequency oscillation suppression strategy incorporating band-stop filtering in the WFMMC voltage sampling is proposed. Compared with the conventional virtual impedance strategy, the proposed strategy reduces the maximum frequency coupling intensity from 2.86 dB to −19.42 dB and improves the phase margin from −45° to 34°, thereby significantly enhancing system stability. Its effectiveness in mitigating frequency coupling and suppressing oscillations under complex conditions, such as increased control delay and active power reference variations, is verified through simulation.
3. The proposed suppression strategy is entirely software-based, requiring only the corresponding digital filter modules within the control algorithm. The filter is inserted immediately after the voltage sampling ADC (Analog-to-Digital Conversion) stage, and is applied exclusively to the raw measurements for protection functions. It necessitates no additional hardware and can be directly integrated into the existing control system of the WFMMC. Filter parameters can be tuned offline based on the impedance characteristics of the actual system. The effectiveness and robustness of the strategy have been validated in a simulation environment, demonstrating strong potential for practical engineering application.

In future work, the feasibility of the proposed strategy in practical engineering applications will be further validated through hardware-in-the-loop simulations and actual hardware experiments in engineering settings.

## Figures and Tables

**Table 1:** Comparison of frequency coupling intensity indicators for WFMMC under V-f control and VSG control.

	WFMMC Adopts V-f Control	WFMMC Adopts VSG Control
Maximum ξ of GSC	−19.01 dB	−1.15 dB
Maximum ξ of WFMMC	−63.54 dB	−2.04 dB

**Table 2:** Comparison of frequency coupling intensity indicators under different WFMMC delays.

	WFMMC Control Delay: 250 μs	WFMMC Control Delay: 450 μs
Maximum ξ of GSC	−23.01 dB	−11.00 dB
Maximum ξ of WFMMC	−5.55 dB	3.24 dB

**Table 3:** Comparison of frequency coupling intensity indicators before and after applying the proposed strategy.

	Before Suppression	After Suppression
Maximum ξ of GSC	−1.15 dB	−19.42 dB
Maximum ξ of WFMMC	−2.04 dB	−19.73 dB

**Table 4:** Comparison of frequency coupling intensity indicators before and after applying the proposed strategy with delay increased to 450 µs.

	Before Suppression	After Suppression
Maximum ξ of GSC	−11.00 dB	−19.61 dB
Maximum ξ of WFMMC	3.24 dB	−18.93 dB

**Table 5:** Comparison of phase margin and frequency coupling intensity indicators between the proposed strategy and virtual impedance.

	Virtual Impedance	Proposed Strategy
Phase Margin	−45°	34°
Maximum ξ of GSC	−4.75 dB	−19.42 dB
Maximum ξ of WFMMC	2.86 dB	−19.73 dB

## Data Availability

The raw data is contained within the article.

## Appendix A

**Table A1:** Electrical parameters of the system.

GSC		WFMMC	
Parameter	Value	Parameter	Value
Rated Power *P*_n,PMSG_ (MW)	700	Rated Power *P*_n,MMC_ (MW)	750
Rated AC Voltage *U*_n,PMSG_ (V)	690	Rated AC Voltage *U*_n,MMC_ (kV)	437
Rated DC Voltage *U*_dc,PMSG_ (V)	1200	Rated DC Voltage *U*_dc,MMC_ (kV)	840
Filter Inductance *L*_f_ (mH)	0.0034	Number of Submodule Units in the Bridge Arm *N*_arm_	500
Filter Capacitance *C*_f_ (F)	0.06	Capacitance of Each Submodule *C*_SM_ (μF)	11,000
Filter Resistance *R*_f_ (Ω)	0.002	Resistance of Single Bridge Arm *R*_arm_ (Ω)	0.1
		Inductance of Single Bridge Arm *L*_arm_ (mH)	140

**Table A2:** Control parameters of the GSC.

Parameter	Value
Proportional Coefficient of PLL PI Controller *k*_p_pll_	43
Integral Coefficient of PLL PI Controller *k*_i_pll_	932
Proportional Coefficient of DC Voltage Loop PI Controller *k*_p_dc_	20
Integral Coefficient of DC Voltage Loop PI Controller *k*_i_dc_	300
Proportional Coefficient of Current Loop PI Controller *k*_p_i_	5
Integral Coefficient of Current Loop PI Controller *k*_i_i_	12

**Table A3:** Control parameters of the WFMMC.

Parameter	Value
Inertia Time Constant *T*_j_	0.036
Damping Coefficient *D*	84.7
Proportional Coefficient of Virtual Excitation PI Controller *k*_pE_	0.2
Integral Coefficient of Virtual Excitation PI Controller *k*_iE_	0.8
Proportional Coefficient of Voltage Loop PI Controller *k*_pu_	2
Integral Coefficient of Voltage Loop PI Controller *k*_iu_	50
Proportional Coefficient of Current Loop PI Controller *k*_pi_	0.8
Integral Coefficient of Current Loop PI Controller *k*_ii_	10
Proportional Coefficient of CCSC PI Controller *k*_pccsc_	1
Integral Coefficient of CCSC PI Controller *k*_iccsc_	20
Control Delay *T*_d_ (μs)	350
